# The Design and Application of Wearable Ultrasound Devices for Detection and Imaging

**DOI:** 10.3390/bios15090561

**Published:** 2025-08-26

**Authors:** Yuning Lei, Jinjie Duan, Qi Qi, Jie Fang, Qian Liu, Shuang Zhou, Yuxiang Wu

**Affiliations:** 1 School of Optoelectronic Materials and Technology, Jianghan University, Wuhan 430056, China; YuningLei@stu.jhun.edu.cn (Y.L.); djjstudy@sina.com (J.D.); QIQI@stu.jhun.edu.cn (Q.Q.); jfang@stu.jhun.edu.cn (J.F.); ShuangZhou@stu.jhun.edu.cn (S.Z.); 2 Institute of Intelligent Sport and Proactive Health, Department of Health and Physical Education, Jianghan University, Wuhan 430056, China; liuqian@stu.jhun.edu.cn; 3 School of Artificial Intelligence, Jianghan University, Wuhan 430056, China

**Keywords:** wearable ultrasound, sensor, continuous monitoring, flexible electronics

## Abstract

The convergence of flexible electronics and miniaturized ultrasound transducers has accelerated the development of wearable ultrasound devices, offering innovative solutions for continuous, non-invasive physiological monitoring and disease diagnosis. This review systematically examines the recent progress in the field, focusing on three key aspects: physical principles, device design, and clinical applications. From the perspective of physical principles, we provide an in-depth analysis of the fundamental theories underlying ultrasound imaging, including acoustic wave propagation in biological tissues, interface reflection mechanisms, and Doppler effects. In terms of device design, we compare technical approaches for rigid and flexible ultrasound transducers, with particular emphasis on innovative designs for flexible transducers. The key developments discussed include optimization of piezoelectric materials, the fabrication of stretchable electrodes, and advances in flexible encapsulation materials. Regarding clinical applications, we categorize the use cases by anatomical region and illustrate their diagnostic value through representative examples, demonstrating their utility in disease detection, health monitoring, and sports medicine. Finally, we identify critical challenges such as signal stability, coupling material compatibility, and long-term wearability, while outlining future directions including AI-assisted diagnosis and multifunctional integration. This review aims to provide a comprehensive reference for both fundamental research and clinical translation of wearable ultrasound technologies.

## 1. Introduction

With the rapid development of modern science and technology and the continuous increase in people’s health awareness, medical monitoring technology is undergoing a revolutionary change [1,2,3]. Although traditional medical ultrasound can provide accurate diagnostic information, its equipment is bulky, complicated to operate, and requires specialized personnel to operate, making it difficult to meet the growing demand for daily health monitoring [4,5]. In this context, wearable ultrasound devices have emerged, opening a new chapter in medical monitoring technology [6,7,8,9].

**Figure 1:**
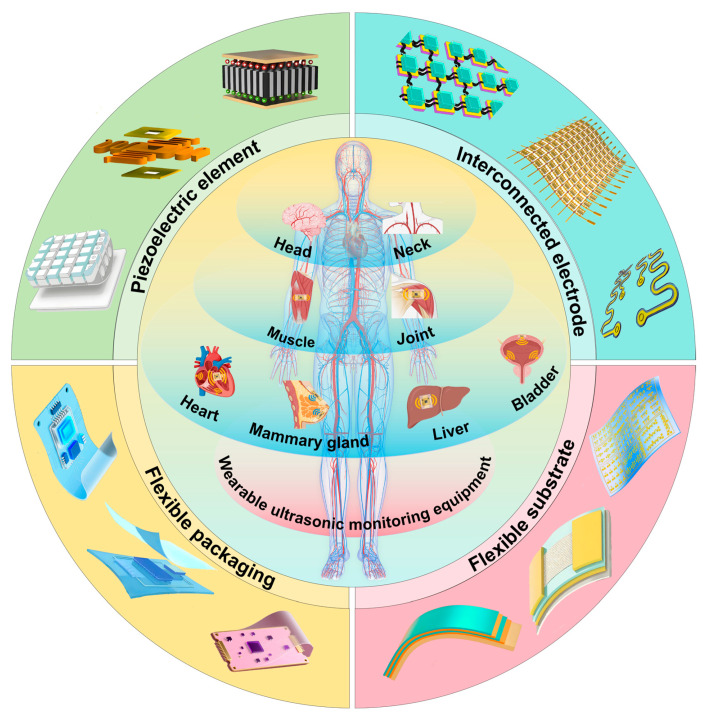
The structural design of wearable ultrasonic transducers and the application of wearable ultrasonic monitoring devices on the human body.

Wearable ultrasound devices are changing the traditional medical-monitoring model with their lightweight, wearable, and easy-to-operate advantages [10,11]. These devices break through the limitations of traditional ultrasound devices, enabling people to monitor their health anytime, anywhere, without the assistance of medical professionals to access key physiological parameters [12,13,14]. During the research and development process, scientists have developed a variety of technical solutions for different application scenarios, ranging from functional devices focused on monitoring hemodynamic parameters such as blood flow velocity and blood pressure to advanced systems capable of visualized imaging of organs and tissues. Although there are also simple-structured and low-power optical sensors that can achieve these detections, such as photoelectric volumetric pulse sensors for heart rate and SpO2, their detection is limited to superficial tissues, generally within a few millimeters beneath the skin surface, and usually does not exceed 1 cm [15]. This is because when light propagates in tissues, it is subject to absorption, scattering, and other effects, and its intensity decays rapidly. Although ultrasound is more complex and has higher power consumption requirements, it performs well in deep tissue imaging and hemodynamic assessment. Commonly used ultrasound probes can reach several centimeters to tens of centimeters beneath the skin. High-frequency probes (such as 7.5–10 MHZ) are typically used for the inspection of shallow structures, and their detection depth may be around 3–5 cm. Low-frequency probes (such as 3.5–5 MHZ) can penetrate deeper tissues, with a detection depth of 10–15 cm or even deeper, and can be used for the examination of deep organs such as the abdomen and pelvic cavity. During abdominal examinations, the maximum scanning depth can reach 20 to 30 cm [13,14]. During a heart examination, the maximum scanning depth is usually around 16 to 24 cm. Thus, wearable ultrasound technology has broad application prospects in the medical and health field [15,16]. In response to the anatomical characteristics and monitoring requirements of different parts of the human body, researchers have designed wearable ultrasound devices with distinctive features [17]. As shown in Figure 1, wearable ultrasound devices now cover all parts of the human body, from cerebral blood flow monitoring in the head to muscle activity tracking in the lower limbs. These devices not only have significant advantages in terms of size and portability but can also ensure the accuracy and reliability of monitoring data through the selection of the appropriate materials and structural design [18,19].

The aim of this paper is to systematically review the latest research progress of wearable ultrasound devices, focusing on their core technology principles, innovative design solutions, and diverse clinical applications. Through in-depth analysis of the existing technologies, we hope to provide new ideas for the future development of flexible ultrasound devices and promote this technology to better serve the field of personalized medicine and health management.

## 2. Principles of Ultrasonic Testing and Imaging

**Figure 2:**
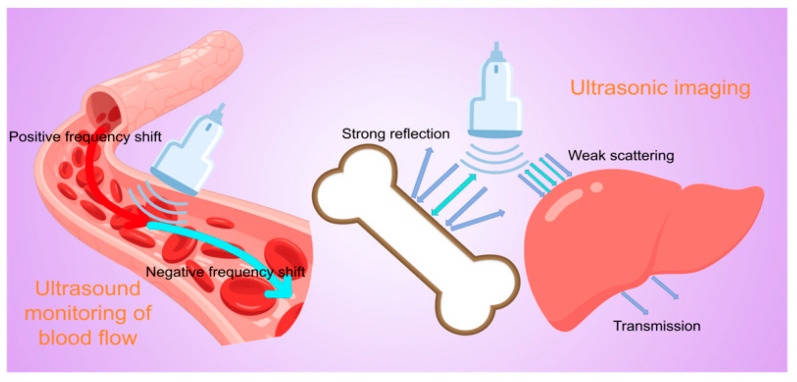
A simplified diagram of the principle of ultrasonic blood-flow monitoring and ultrasonic organ imaging.

The principle of ultrasonic blood flow detection and organ imaging is based on the propagation characteristics of high-frequency sound waves in biological tissues and their interactions with different media [20,21,22]. Combined with modern signal-processing techniques, it enables non-invasive visualization of internal structures and hemodynamics in the human body. The core physical foundation lies in the reflection and scattering of sound waves at tissue interfaces, as well as the Doppler frequency shift effect caused by moving red blood cells [23,24,25] (Figure 2).

The detection of blood flow by ultrasound relies heavily on the physical phenomenon of the Doppler effect. When ultrasound encounters flowing blood, the erythrocytes act as scatterers to shift the frequency of the sound wave by an amount proportional to the velocity of the erythrocyte motion, and in a direction that depends on the direction of motion of the blood flow with respect to the probe. Specifically, blood moving toward the probe compresses the wavelength of the reflected sound wave, resulting in an increase in frequency (positive frequency shift), while blood moving away from the probe stretches the wavelength of the sound wave, resulting in a decrease in frequency (negative frequency shift). This frequency shift can be extracted and analyzed by signal-processing techniques such as fast Fourier transform [26,27]. In clinical applications, color Doppler imaging color-codes the frequency shift information (usually using red and blue colors to indicate blood flow in opposite directions) and superimposes it on the anatomical images of B-mode ultrasound in real time, allowing physicians to visualize the spatial distribution of and directional changes in intravascular blood flow. On the other hand, Spectral Doppler displays the changes in blood flow velocity over time at a certain point or line as a spectral waveform through pulsed-wave or continuous-wave sampling, providing quantitative parameters such as peak systolic flow velocity, end-diastolic flow velocity, resistance index, etc., which are important for the assessment of vascular stenosis, valvular function, etc. [28,29,30,31]. In addition, the energy Doppler technique significantly improves the sensitivity of detecting low-velocity blood flow and microvessels by detecting the energy rather than the frequency change in the scattered signals, which provides a unique advantage in observing organ perfusion and tumor angiogenesis [32,33].

During the imaging process, the ultrasound probe emits pulsed high-frequency sound waves (usually in the range of 2–20 MHz depending on the depth of the examined area and the resolution required) into the body, and these sound waves are reflected to varying degrees due to differences in the acoustic impedance of the tissues as they propagate through the body [34,35,36]. Interfaces with greater differences in acoustic impedance (e.g., between soft tissue and bone) show stronger reflections, whereas homogeneous tissues (e.g., liver parenchyma) or liquids (e.g., blood and bile) show mainly transmission or weak scattering of sound waves [37,38,39]. By accurately measuring the return time (time-of-flight method) and intensity of the reflected sound waves detected by the receiving probe, combined with the average value of the velocity of sound in human tissues (about 1540 m/s), the spatial location of each reflective interface can be calculated, and then this point information can be synthesized into a two-dimensional or even three-dimensional tomographic image by means of electronic scanning technology, which is the basic principle of B-mode ultrasound imaging [40,41,42].

Modern ultrasound devices also incorporate a variety of advanced techniques to improve imaging quality. For example, harmonic imaging utilizes high harmonic components generated by tissue nonlinear effects to effectively suppress noise interference from superficial tissues [43,44]; spatial composite imaging reduces anisotropic artifacts by emitting beams from multiple angles; and elastography evaluates the stiffness of tissues by detecting their deformation properties under mechanical excitation, which provides diagnostic information for pathologies such as hepatic fibrosis and breast lumps [45,46,47]. For hemodynamic analysis, the latest vectorial flow imaging techniques break through the angle-dependent limitations of conventional Doppler and can more accurately display complex blood flow patterns such as eddy currents and turbulence [48,49]. The combined application of these techniques enables modern ultrasound to not only clearly display fine anatomical structures, from superficial blood vessels to deep organs, but it also allows for the assessment of physiological changes in tissue perfusion and vascular function, even at the cellular level in real time and dynamically, which plays an irreplaceable role in a number of clinical areas, such as the diagnosis of cardiovascular diseases, tumor screening, and obstetrical monitoring [50,51,52].

## 3. Design and Fabrication of Wearable Ultrasonic Transducers

**Figure 3:**
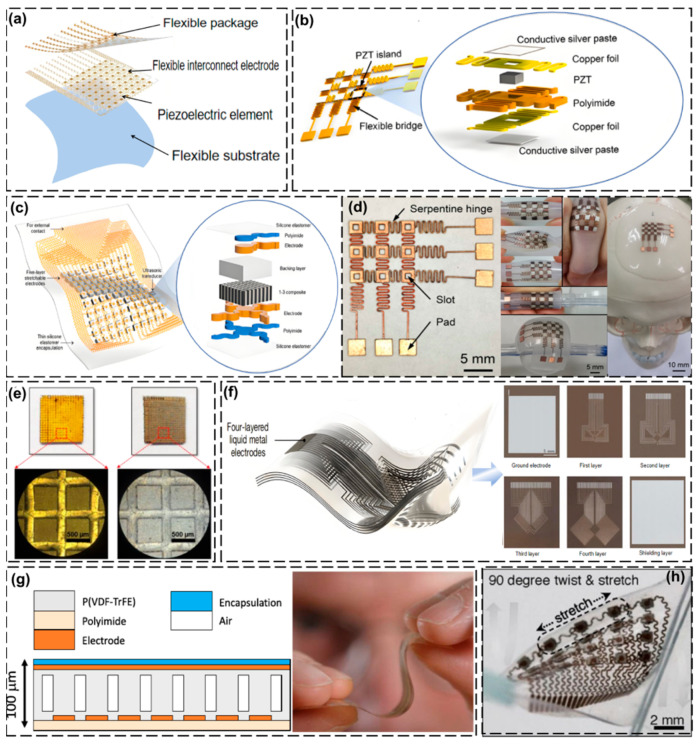
Design and fabrication of wearable ultrasonic transducers. (**a**) Flexible ultrasonic transducer structure. Reproduced with permission: Copyright 2020, Springer Nature [71]. (**b**) Three-dimensional schematic of array structure and its partial exploded view. Reproduced with permission: Copyright 2021, IEEE [72]. (**c**) Scalable ultrasonic imaging array for three-dimensional imaging of complex surfaces. Reproduced with permission: Copyright 2018, Science Advances [73]. (**d**) Laser-ablated flexible hinges with addressable electrode arrangement and conforming to a 1:1 human skull model. Reproduced with permission: Copyright 2021, IEEE [72]. (**e**) Gold electrode and Au/AgNW electrode. Reproduced with permission: Copyright 2020, IEEE [74]. (**f**) Multilayer liquid metal composite electrode. Reproduced with permission: Copyright 2023, Springer Nature [75]. (**g**) Schematic cross section of flexible ultrasound transducers and photograph of finished ultrasound transducer foil. Reproduced with permission: Copyright 2024, Springer Nature [76]. (**h**) Hybrid composite electrode made of hydrogel and elastomer. Reproduced with permission: Copyright 2018, Springer Nature [77].

In medical ultrasound detection, the transducer is an important component, and the materials currently used in ultrasound transducers are generally selected from piezoelectric materials, such as piezoelectric ceramics and piezoelectric composites, which are usually rigid [53,54,55,56]. The designs for wearable ultrasound devices are broadly divided into two categories: one category uses a traditional rigid transducer tied to the user’s body using straps or elastic bands [57,58,59], and the other uses flexible transducers attached to the user’s body in the form of patches. Flexible patch transducers can closely adhere to the skin, which makes the acquired signal more stable, and the transducers are more compact and comfortable for the user [60,61,62,63]. This structural innovation has attracted the interest of many scholars, so we will focus on the design and preparation of flexible patch transducers, but we will also introduce some examples of strap-on rigid transducers in the following practical application examples. The implementation of flexible piezoelectric arrays fundamentally relies on their architectural integration with compliant substrates—either through assembly on bendable (non-stretchable) polymer platforms or through direct fabrication on elastomeric stretchable substrates [64,65,66,67]. Local rigidity and overall flexibility are combined in order to make the transducer flexible; the elements are connected through interconnecting electrodes and further encapsulated with flexible materials so that the whole device can be bent or stretched while satisfying the required acoustic performance and realizing the combination of local rigidity and overall flexibility [68,69]. Flexible ultrasonic transducers are mainly composed of four parts: a flexible substrate, piezoelectric elements, flexible interconnecting electrodes, and flexible encapsulation [70,71] (Figure 3a). A summary is shown in Table 1.

### 3.1. Piezoelectric Elements

In flexible transducers, the piezoelectric elements are usually in a multi-array element structure, which requires that each individual piezoelectric element can meet the ultrasonic transmission standards, which depends on the material and fabrication process, and the multiple arrays composed of piezoelectric elements need to be coordinated with each other without crosstalk [102,103]. Earlier research has demonstrated that transducer flexibility can be achieved through the deposition of piezoelectric thin films (e.g., zinc oxide [ZnO] and aluminum nitride [AlN]) onto flexible substrates [80,81,82]. However, the piezoelectric constants of ZnO (d33, f of 5.9 pm/V) and AlN (d33, f of 3.9 pm/V) are much lower than those of conventional lead zirconium titanate (PZT) piezoelectric ceramics [78,79]. In addition, piezoelectric polymers such as polyvinylidene fluoride (PVDF) and its copolymers have been used to fabricate flexible transducers, but their suitability for transmitting ultrasound is limited due to their low electromechanical coupling coefficients and dielectric constants [104,105,106,107,108].

Liu et al. [72] proposed an innovative flexible and stretchable ultrasonic transducer array with a 3 × 3 element structure that is encapsulated with an elastomer (Figure 3b). This electrode layout design enables each component to be excited through a small number of connecting wires. The developed transducer has a spatial pulse length of approximately 10 µs and a bandwidth (−6 dB) of about 9.8% in pulse-echo testing at a resonant frequency of 1.91 MHz, demonstrating excellent acoustic characteristics.

In practical diagnostic applications, the array element spacing needs to be small enough to minimize crosstalk between the array elements, yet sufficient space must be left for interconnecting electrodes to ensure that the device is stretchable. The stretchable ultrasonic transducer array developed by Hu et al. [73] has a 10 × 10 element structure and adopts an “island-bridge” layout. The spacing between adjacent array elements is 2.0 mm, and the size of a single array element is 1.2 mm × 1.2 mm, with a spacing of 0.8 mm. The base and covering layer are 15 μm thick silicone rubber elastomer films. The overall thickness of the equipment is relatively thin, with a stretchability exceeding 30%, while minimizing grating artifacts in the image, as shown in Figure 3c. This array exhibits a mean resonant frequency of 3.51 MHz with a standard deviation of 56.8 kHz and a mean antiresonant frequency of 4.30 MHz with a standard deviation of 59.1 kHz, demonstrating good uniformity across all 100 functional elements.

### 3.2. Interconnected Electrodes

Flexible interconnecting electrodes are crucial for piezoelectric element integration, which requires both high conductivity under mechanical stress and an optimal balance between flexibility and charge transport [109,110]. This property is both an advantage and a challenge for innovation. Currently, there are two main material options for flexible interconnect electrodes: solid metals and liquid metals [111,112,113].

Solid metals are the material of choice for electrodes due to their excellent electrical conductivity, and a certain degree of stretchability can be achieved by adjusting the geometry. The most commonly used design is the serpentine wire bridge structure, which is effective in enhancing ductility in flexible electronic devices [83,84,85,86]. These electrodes are typically fabricated using micro/nanofabrication techniques including lithography, 3D printing, and screen printing. Polymeric substrates such as polyimide (PI) or polyethylene terephthalate (PET) are commonly employed to provide mechanical support and improve metal–elastomer interfacial adhesion [90,91].

Liu et al. [72] used commercial PZT (lead zirconate titanate) as the piezoelectric material in their research. The spacing between the rigid PZT islands was designed to be 4.5 mm, and the size of each PZT block was 1 mm × 1 mm × 0.5 mm (thickness). The designed electrodes adopted a double-sided conductive serpentine hinge structure for electrical connection (Figure 3d), an addressable configuration in which the electrodes operate by activating specific array units while maintaining a single layer of electrical interconnections, with the aim of efficiently connecting multiple array elements and reducing the number of wires. In addition, the electrodes are fabricated using laser precision machining to directly form a serpentine hinge and fix the PZTs with conductive silver paste, thus ensuring a good electrical connection. The design not only excels in electrical performance, but also maintains mechanical integrity even when uniaxially stretched up to 33%.

Although solid metal electrodes enhance the flexibility of a device, they can still fracture when stretched or bent, limiting their flexibility and stretchability. The tensile limit of solid metals (such as Cu serpentine electrodes) is approximately 30–50%. Beyond this range, a sudden increase in resistance (>100%) will occur [114]. In contrast, liquid metal electrodes such as silver nanowires (AgNWs) and eutectic gallium–indium (EGaIn) exhibit better stretchability. The resistance change rate of a AgNW network is less than 20% when the stretchability rate is 80%, but it fails due to nanowire breakage when the stretchability exceeds 100% [87]. The resistance fluctuation of an EGaIn liquid metal electrode is only less than 5% at a tensile rate of 500% [88]. AgNWs have a relatively high electrical conductivity, with an electrical conductivity typically reaching around 10^7^ S/m. The electrical conductivity of EGaIn is approximately 3.4 × 10^6^ S/m. Although it is lower than that of AgNWs, its electrical conductivity is superior among liquid materials and can meet the electrical conductivity requirements of many electronic devices. AgNWs are prone to oxidation in air, with an oxide layer forming on its surface, which can affect their electrical conductivity and other properties. Usually, packaging or surface treatment is required to enhance stability. In air, EGaIn rapidly forms a layer of gallium oxide film on its surface. This film can protect the internal metal and give it good chemical stability [87,88]. However, in some special environments, such as strongly acidic ones, chemical reactions may still occur. AgNWs have certain biocompatibility and antibacterial properties, and can be applied in biosensors, etc. However, silver ions may be cytotoxic. Gallium and indium have good biocompatibility and have been proven to be suitable for monitoring animal brains. They can accurately record neural signals by closely adhering to the cortical surface and are suitable for flexible bioelectronic devices, such as implantable bioelectronic devices. Fabricating electrodes by depositing AgNWs or EGaIn on a flexible substrate and curing it to form a permeation network produces electrodes with extremely high electrical conductivity and extensibility [89,115].

Kim et al. [74] coated the surface of PZT-5H with AgNWs using spin-coating technology. The width of the PZT-5H piezoelectric column was less than 0.5 mm and the kerf width (the spacing between adjacent PZT columns) was 0.15 mm, achieving a ceramic volume fraction of 60%. The whole column could be bent to a curvature radius of less than 5 mm. The resonant frequency of this transducer was approximately 1.7 MHz, and the acoustic impedance corresponding to the anti-resonant frequency was calculated to be 19.2 MRayl. The −6 dB fractional bandwidth (FBD) was approximately 49%, and the transmission sensitivity was about 107 mV/V. In the pulse excitation test, effective ultrasonic emission and reception were achieved when the input pulse energy was 8 μJ, indicating a relatively high energy conversion efficiency (Figure 3e). Hu et al. [75] developed high-density multilayer stretchable electrodes based on EGaIn for wearable cardiac ultrasound imaging devices, which, in addition to maintaining excellent electrical conductivity and stretchability, possessed a certain degree of bioadhesion and enhanced the coupling effect between the device and the skin (Figure 3f).

### 3.3. Flexible Substrate

The elasticity and stability of the substrate materials are key factors affecting the performance of flexible transducers; these materials also need to be non-toxic and non-irritating to ensure comfort when worn [116,117]. Common base materials include polydimethylsiloxane (PDMS), aliphatic/aromatic copolyesters (Ecoflex), hydrogels, polyurethanes (PUs), as well as some conductive polymers and nanomaterials (e.g., carbon nanotubes and graphene) as reinforcement materials [96,97]. Among them, Ecoflex and PU provide good deformability, while hydrogels are favored for their superior biocompatibility. PDMS is ideal for flexible substrate materials due to its good biocompatibility, non-toxicity, resistance to oxidization, high flexibility, high coefficient of thermal expansion, and high transparency [92,93]. However, PDMS has poor adhesion when combined with solid materials, making it difficult to realize effective coupling. To improve this problem, the surface adhesion of PDMS can be enhanced through UV irradiation, oxygen plasma treatment, or the addition of non-ionic surfactants [94,95]. In terms of fabrication processes, inkjet printing, flexible circuit board technology, microfabrication, laminate technology, and dip-coating are widely used for the production of substrate materials.

Neer et al. [76] proposed a piezoelectric polymer thermos imprint-based ultrasound-on-foil transducer technology, Pillar Wave, which they used to laminate approximately 40 um thick P(VDF-TrFE) films onto a polyimide substrate (14 um thick) containing a thin bottom electrode. This P(VDF-TrFE) film was structured by thermal embossing with a PDMS impression, and a uniform columnar structure with a height of 70 μm was formed on top of a residual 10 μm thick P(VDF-TrFE) film. After the imprinting step, a second layer of an ~10 um thick P(VDF-TrFE) film was laminated on top to provide a flat surface for the deposition of the patterned top electrodes. This P(VDF-TrFE) (lead-free) film has good biocompatibility. The encapsulation material was a flexible isolation layer (such as polyimide, parylene C), which is non-toxic and non-irritating, and is suitable for direct contact with human skin (Figure 3g).

### 3.4. Flexible Packaging

Compared with traditional transducer structures, flexible ultrasound transducers use a soft film instead of the traditional metal shell to encapsulate the piezoelectric element, allowing for a close fit between the device and the skin. Because the transducer is in direct contact with the skin, the acoustic impedance of the substrate material needs to match the acoustic impedance of skin, so the substrate is usually prepared using hydrogels, organic elastomers, or mixtures of the two, which are non-toxic and non-irritating and can also effectively realize encapsulation and acoustic coupling functions [100,101].

However, a problem with conventional hydrogels is that their wettability cannot be maintained for a long time after encapsulation, and after drying, the hardness increases the density and the acoustic impedance of the hydrogel changes, which affects acoustic wave propagation and leads to a loss of adhesion [98,99]. To address this challenge, Wang et al. [77] developed a hybrid composite material consisting of hydrogel and elastomer, with a thickness of only 240 μm. It combines the advantages of softness, toughness, anti-dehydration, and bioadhesion. The modulus is close to that of skin (about 5 kPa) and it has excellent biocompatibility. The survival rate of fibroblasts (HF-1) after continuous ultrasound irradiation was 100%. It could firmly adhere to the skin for up to 48 h. This composite material could not only effectively replace the traditional coupling agent, enhancing the transmission efficiency of sound waves to the tissue, but also provide protection for piezoelectric components (Figure 3h).

## 4. Application of Wearable Ultrasonic Monitoring Devices

In the above sections, we introduced the design and preparation of some flexible wearable ultrasound transducers. Next, this paper introduces some specific application scenarios of wearable ultrasound monitoring devices from the perspective of practical applications, from the assessment of cerebrovascular hemodynamics in the head and neck region, to the precise imaging of cardiac function and breast tissues in the thoracic cavity, to monitoring the elasticity and morphology of the abdominal cavity organs (such as the liver and bladder) and the vascular blood pressure and musculoskeletal biomechanics in the limbs. Wearable ultrasound technology has redefined the boundaries of clinical diagnosis and health management. Its lightweight, flexible, and wireless design concepts not only enhance patient comfort and compliance, but also provide a scientific basis for early disease warning and personalized treatment decision-making through continuous data acquisition and cloud-based analysis [118,119]. A summary is shown in Table 2.

### 4.1. Head and Neck Area

**Figure 4:**
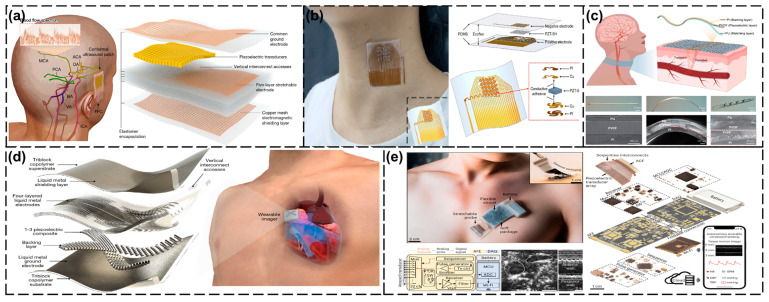
Wearable ultrasound testing in the head, neck, and chest. (**a**) Schematic of the configuration and structure of a patch. Reproduced with permission: Copyright 2024, Springer Nature [120]. (**b**) Overall structure of a sensor and the structure of a single array element. Reproduced with permission: Copyright 2024, IEEE [121]. (**c**) Schematic of the design and working principle of the PUT. Reproduced with permission: Copyright 2024, Springer Nature [122]. (**d**) Schematic showing the exploded view of a wearable imager. Reproduced with permission: Copyright 2023, Springer Nature [75]. (**e**) The fully integrated USoP. Reproduced with permission: Copyright 2023, Springer Nature [123].

Intracranial blood flow monitoring has an important role in clinical neurocritical care and basic neurovascular research. By accurately and continuously monitoring cerebral blood flow, brain diseases can be effectively screened and diagnosed [132]. In this field, Zhou et al. [120] made an important breakthrough with the development of a new ergonomic ultrasound patch. The patch utilizes low-frequency ultrasound (2 MHz) to penetrate different cranial windows (temporal, orbital, submandibular, and suboccipital windows) to achieve three-dimensional volumetric imaging of the cerebral arterial network and continuous recording of blood flow spectra in selected arterial segments. This technique has significant advantages over conventional TCD probes. Firstly, the low-frequency ultrasound with copper mesh shielding improves the signal-to-noise ratio by about 5 dB. Although the current design has a relatively low spatial resolution, making it impossible to obtain information about tiny blood vessels and the post-processing time for volume image reconstruction is long, which limits the ability to perform real-time dynamic monitoring, it can effectively overcome the problems of signal attenuation and phase distortion caused by the skull. Secondly, the stable contact characteristic of the patch avoids the signal fluctuation problem caused by head movement that traditional probes face, which provides a reliable guarantee for long-term monitoring. The author compared the data of traditional TCD probes and conformance ultrasound patches of 36 participants. It was found that the differences between the two devices in peak contraction velocity, average flow velocity and end-diastolic velocity were very small (−1.51 ± 4.34 cm s^−1^, −0.84 ± 3.06 cm s^−1^, and −0.50 ± 2.55 cm s^−1^, respectively). This indicates that the conformance ultrasound patch can produce comparable accuracy to that of traditional TCD probes, thereby verifying its ability to continuously monitor cerebral blood flow. All the studies in this experiment were approved by the Institutional Review Board of the University of California, San Diego, and are registered on ClinicalTrials.gov. The safety of ultrasound exposure was thoroughly evaluated to ensure that it did not exceed the maximum level recommended by the FDA to ensure the safety of the participants (Figure 4a).

In the field of cardiovascular monitoring, the continuous monitoring of arterial blood pressure (ABP) is an important risk assessment indicator [133]. Liu et al. [121] achieved a breakthrough with the development of a flexible and stretchable ultrasound transducer array (39 mm × 37 mm × 0.71 mm). The equipment uses PZT-5H materials and a frequency of 4 MHz. Through simulation and optimization design, it was designed into a 5 × 5 array, with each array element having a size of 1.6 mm × 1.6 mm and a spacing of 1 mm. This device not only seamlessly adheres to the skin for non-invasive detection, but its phased array focused emission mode also ensures the accuracy of carotid artery blood-pressure monitoring. The Complior central artery pressure detector that has been put into use in hospitals was used for comparison. The blood pressure measurement results at four different time points showed that the average relative errors were 6.14%, 5.89%, 6.15%, and 7.00%, indicating that the measurement results of the flexible array have high reliability and have been clinically verified to be consistent with the results of the standard detector. Although this technology overcomes the limitations of the traditional cuff method for intermittent measurements and invasive detection of ductus arteriosus, the stability of the flexible transducer array needs to be further improved to enable the algorithm to accurately extract the positions of the anterior and posterior walls of the blood vessel when processing echo data (Figure 4b). In addition, the braidable polymer ultrasound transducer (PUT) developed by Zou et al. [122] demonstrated excellent performance. Its 93% ultra-wide bandwidth (3.68–10.08 MHz) allowed it to adapt to a wide range of tissue-monitoring needs, and its performance remained stable even after 10,000 deformation tests. This device can be seamlessly integrated into textiles for real-time cardiovascular monitoring, and the potential applications of PUT include tissue and vascular monitoring at multiple frequencies. In a carotid artery blood flow velocity monitoring experiment, volunteers wore sweaters woven with PUT fibers and their carotid artery blood flow velocity was detected using a 10 MHz single-frequency continuous wave. The obtained blood flow spectrum characteristics were similar to those of commercial ultrasound probes tested in the neck, indicating that the signal-to-noise ratio of PUT in this application scenario can reach a comparable level to that of commercial probes (Figure 4c).

In summary, the new ultrasound monitoring technology for the head and neck area has significant advantages over traditional equipment: in terms of intracranial blood flow monitoring, it solves the problem of the unstable signals caused by skull attenuation and movement interference of traditional TCD probes, and realizes three-dimensional continuous imaging of cerebral blood flow. In terms of cardiovascular monitoring, the flexible ultrasound transducer array achieves non-invasive continuous blood pressure monitoring through phased array focusing, avoiding the intermittent limitations of the cuff method and the invasive risks of ductus arteriosus. The braided polymer transducer, with its ultra-wide bandwidth and ten-thousand-cycle deformation stability, breaks through the rigidity limitations of traditional equipment while maintaining a signal-to-noise ratio comparable to that of commercial probes.

### 4.2. Thoracic Cavity

The heart is certainly at the top of the list of the organs of greatest concern in the thoracic region. Continuous cardiac-function monitoring is crucial for the early detection and intervention in cardiovascular diseases, but conventional non-invasive imaging devices are bulky and cannot easily achieve dynamic monitoring [134]. To address this challenge, Hu et al. [75] developed a wearable cardiac ultrasound imaging device. The device utilizes a piezoelectric transducer array, liquid metal composite electrodes, and a triblock copolymer package. The structure adopts an orthogonal geometric design, similar to a Mills cross array, to achieve simultaneous acquisition of standard views of two planes. Each transducer array consists of four arms, each of which has six small elements combined in a row to form a long element, and a central section shared by the four arms. The number of components in each direction is 32, and the distance between the components is 0.4 mm. The transducer uses 1–3 composite materials for the emission and reception of ultrasonic waves. The acoustic impedance of the 1–3 composite materials is close to that of the skin, maximizing the propagation of acoustic energy in human tissues and significantly enhancing the mechanical coupling performance between the device and the skin, thereby supporting real-time monitoring of left ventricular function from multiple angles. The device integrates deep learning algorithms, enabling the system to automatically analyze continuous images and calculate key cardiac indicators (such as stroke volume, cardiac output, and ejection fraction) in real time. This overcomes the limitations of traditional devices, which are bulky and unable to monitor continuously. This device also has some issues that need to be improved. For instance, although it can adapt to the static human chest, advanced imaging algorithms need to be further developed for use in dynamic situations to compensate for phase distortion. At present, the equipment is connected to the back-end system through flexible cables for data processing. In the future, further miniaturization and integration are needed. Deep learning models are currently only applicable to the subjects in the training dataset, and their generalization ability needs to be improved by expanding the training dataset or optimizing the network structure. In addition, combined with deep learning algorithms, the system can automatically analyze continuous images and calculate key cardiac metrics (e.g., output per beat, cardiac output, and ejection fraction) in real time, overcoming the limitations of traditional devices that are bulky and unable to monitor continuously (Figure 4d).Lin et al. [123] have further optimized this technique by proposing a fully integrated wearable ultrasound system (USoP), which can be used for motion continuous monitoring of central blood pressure, heart rate, and cardiac output for up to 12 h and during exercise. The system utilizes M-mode imaging, which allows stable tracking of carotid artery pulsations even during movements such as head rotation. In cycling and high-intensity interval training (HIIT) experiments, USoP successfully recorded the dynamic changes in the participants’ hemodynamics. All the experiments were approved by the Institutional Review Board (IRB) of the University of California, San Diego, and all the participants signed informed consent forms and voluntarily participated in the experiments. Although this device could be a new tool for sports medicine and cardiovascular monitoring and has great potential, it also has some problems. For instance, when the soft ultrasound probe is attached to dynamic and curved skin surfaces, the unknown position of the transducer may lead to phase distortion and a decline in B-mode imaging quality. To solve this problem, it may be necessary to apply additional shape sensors or develop iterative comparison optimization algorithms. In addition, the long-term wearing performance requires further enhancement, for instance, by integrating multi-layer soft circuits, combining wearable energy harvesting devices, and using more durable and breathable adhesives (Figure 4e).

**Figure 5:**
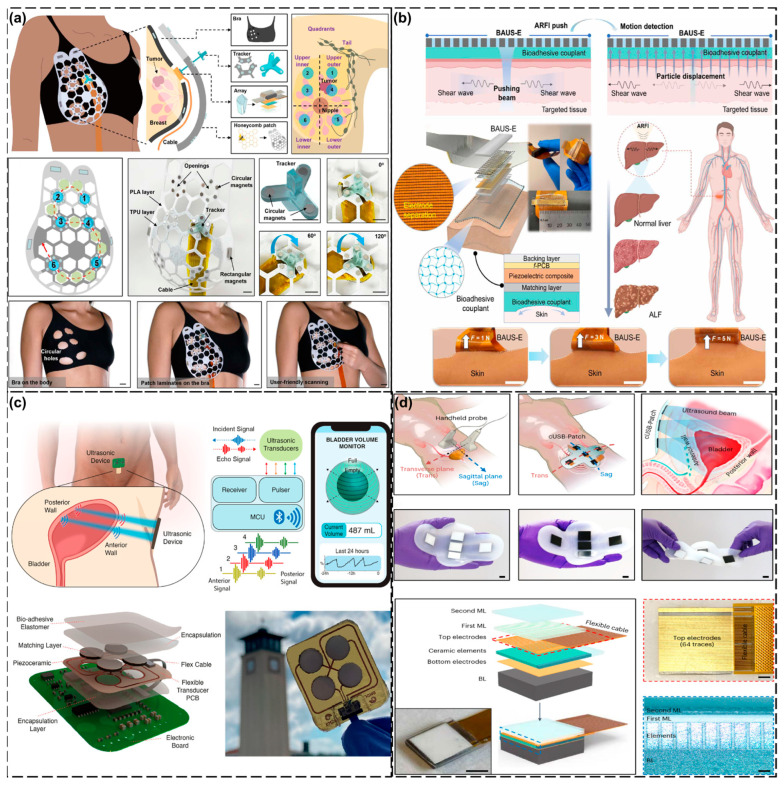
Wearable ultrasound testing in the chest and abdomen. (**a**) Ultrasound breast patch used for deep tissue scanning and imaging. Reproduced with permission: Copyright 2023, Science Advances [124]. (**b**) Design and mechanism of wearable BAUS-E. Reproduced with permission: Copyright 2024, Science Advance [125]. (**c**) Design and working principle of integrated ultrasonic device. Reproduced with permission: Copyright 2024, Springer Nature [126]. (**d**) Overview of phased array and cUSB-Patch. Reproduced with permission: Copyright 2023, Springer Nature [127].

Another very important organ in the chest area for women is the mammary gland. Whenever people talk about the mammary glands, they often mention another phrase: breast cancer. Early screening of breast cancer is crucial to increase the cure rate. Conventional ultrasound relies on manual manipulation, which makes it difficult to achieve standardized and reproducible imaging [135]. For this reason, Du et al. [124] developed a flexible wearable ultrasound breast patch (cUSBr-Patch) to support large-area, deep-tissue scanning and multi-angle imaging. The patch utilizes an Yb/Bi-doped PIN-PMN-PT single-crystal phased array that exhibits a wider field of view than commercial linear probes at a depth of 30 mm and has a reduced reliance on operator experience. However, the research on this device is currently mainly at the laboratory and preliminary clinical trial stages, and large-scale clinical verification has not yet been carried out. Moreover, the design and manufacturing costs of this patch are relatively high, which may limit its wide application. Therefore, large-scale clinical verification in later stages may encounter difficulties (Figure 5a).

In summary, in the field of thoracic health monitoring, wearable ultrasound technology has made breakthroughs and has advantages over traditional devices: flexible cardiac ultrasound devices use piezoelectric arrays and liquid metal electrodes, combined with deep learning algorithms to achieve real-time dynamic monitoring of left ventricular function (such as ejection fraction), solving the pain points of traditional ultrasound devices as they are bulky and unable to record continuously. Through M-mode imaging, hemodynamic changes can be stably tracked during motion, overcoming the limitation of traditional monitoring, which is disturbed by motion. For breast screening, flexible ultrasound patches using doped single-crystal phase-controlled arrays can achieve large-field imaging at a depth of 30 mm. Its standardized scanning mode overcomes the problem that traditional ultrasound relies on the operator’s experience.

### 4.3. Abdominal Cavity

Our abdominal cavity is home to a number of organs that play roles in our vital activities, but it is also a disease-prone area for a variety of reasons. Acute Liver Failure (ALF) is a clinically critical condition characterized by rapid progression to hepatic encephalopathy within 8 weeks of the first symptoms in patients with no previous history of chronic liver disease. The etiology of the disease is complex and varied, mainly including viral hepatitis, pharmacologic liver injury, circulatory failure, and other causative factors [136]. Because of the rapid progression of ALF and its poor prognosis, with a mortality rate of up to 80%, early diagnosis and timely intervention are essential. Liu’s research team [125] reported a breakthrough technology, Bioadhesive Ultrasound Shear Elastography (BAUS-E), which is a new technology for the detection of ALF. Integration of this innovative technology into a wearable device allowed for continuous tracking of disease progression in a rat ALF model through real-time monitoring of the dynamic changes in liver stiffness. By comparing the liver histopathological staining results (such as HE staining, Sirius red staining, and α-SMA staining) at different time points, a correlation between the changes in liver stiffness measured by BAUS-E and the progression of ALF was verified. Furthermore, through regression analysis, it was confirmed that the Young’s modulus measured by BAUS-E was highly correlated with the results of the Sirius red staining (R = 0.94, *p* < 0.001) and α-SMA staining (R = 0.91, *p* < 0.001). All the experiments were conducted in accordance with the guidelines and protocols approved by the Institutional Animal Care and Use Board (IACUC) of the University of Southern California (USC). The experimental data confirmed that the BAUS-E system can accurately capture the changes in liver mechanical properties during the course of ALF, demonstrating its significant early diagnostic value and potential for prognosis evaluation and providing an important non-invasive monitoring method for the clinical management of ALF. A limitation for the current BAUS-E device is that it needs to be connected to the Verasonics Vantage research ultrasound system, which limits its portability and range of clinical applications. Future research should focus on integrating external power supplies and data processing into chips to produce integrated portable devices (Figure 5b).

Lower Urinary Tract Dysfunction (LUTD) is a class of clinical syndromes involving abnormalities in bladder storage and voiding function and has a global prevalence of approximately 2.3 billion, which is expected to continue to climb with the accelerated aging of the population [137]. The pathogenesis of this condition is complex, with the main causes including benign prostatic hyperplasia (BPH) and neurogenic bladder (e.g., due to multiple sclerosis, spinal cord injury, and other neurologic disorders) [138]. In the clinical management of LUTD, accurate bladder volume monitoring has a critical diagnostic value and is an important basis for assessing the functional status of the bladder and for formulating treatment strategies. In recent years, wearable ultrasound technology has made significant breakthroughs in the field of bladder-function monitoring. The integrated flexible ultrasound bladder volume monitoring system (Ultrasound Bladder Volume Monitor, UBVM) developed by the Toymus research team [126] utilizes multipoint ultrasound reflectance signal acquisition technology combined with advanced spherical fitting algorithms to realize continuous wireless monitoring of bladder volume. In vitro experiments using this system were conducted by simulating bladders of different shapes and sizes in a water tank and using flasks of various shapes to evaluate the accuracy of the equipment. For simulated bladders of different shapes and volumes (such as round-bottomed flasks), the average relative error was 14.85%. In the in vivo experiment, practical application tests were conducted on five healthy volunteers (all of whom had signed informed consent forms and undergone health status and medical history investigations) to verify the performance of the device in a real environment. For bladder volumes within the range of 100 to 800 mL, the average relative error was 11.17% (allowable error: ≤15%) [139,140]. In addition, compared with traditional ultrasound imaging methods, the volume estimation results of the UBVM equipment were highly consistent. Linear regression analysis showed that the R-squared value was 99.5%, meeting the clinical diagnostic requirements. At present, the number of sensors in the equipment is limited. Increasing the number of sensors may improve the measurement accuracy, especially when dealing with bladders with complex shapes (Figure 5c).More notably, the Conformable Ultrasound Bladder Patch (cUSB-Patch) developed by Zhang’s team [127] has an even higher monitoring accuracy. The device utilizes a unique five-array design that enables real-time imaging from multiple angles without the need to move or rotate the probe, which is required in conventional ultrasound examinations. The samarium/lanthanum-doped PMN-PT ceramic material used in this device has superior piezoelectric performance. Compared with traditional materials (such as PZT-5H), this new material can significantly increase the bandwidth and detection depth of ultrasonic equipment, providing new possibilities for future electromechanical applications. In addition, this material also demonstrates better temperature stability, which helps to enhance the reliability and durability of the equipment in various environments. Twenty subjects were recruited for bladder volume monitoring in the experiment. The clinical test results showed that regardless of whether ultrasound coupling agents were used or not, the volume measurement error could be controlled within the ideal range (3.2 ± 6.4% when using coupling agents and 10.8 ± 8.2% without coupling agents), and its performance indicators reached the detection level of conventional clinical ultrasound equipment. However, the long-term stability and reliability of the cUSB-Patch have not been fully verified, and these characteristics will need to be evaluated in the future (Figure 5d).

In summary, in the field of abdominal-organ monitoring, wearable ultrasound technology has demonstrated significant advantages over traditional devices: for the monitoring of acute liver failure, the bioadhesive ultrasound elastography system has achieved continuous non-invasive early diagnosis, which is difficult to achieve with traditional methods, through real-time tracking of liver stiffness changes. For lower urinary tract dysfunction, the flexible bladder monitoring system adopts multi-point reflection signals and spherical fitting algorithms to control the bladder volume measurement error within 11.17%. The adaptive phased array patch further can achieve a lower error of 3.2% due to its five-array design (using coupling agents), and can achieve multi-angle imaging without the need for probe movement, which is needed in traditional ultrasound imaging. In the field of abdominal imaging, dynamic and continuous monitoring of organ functions has been achieved (overcoming the intermittency of traditional examinations), enhancing operational convenience (breaking away from the reliance on professional operations) and improving measurement accuracy (reaching clinical ultrasound standards). In particular, it has unique clinical value in providing early warnings of liver failure and long-term tracking of bladder function.

### 4.4. Limbs

**Figure 6:**
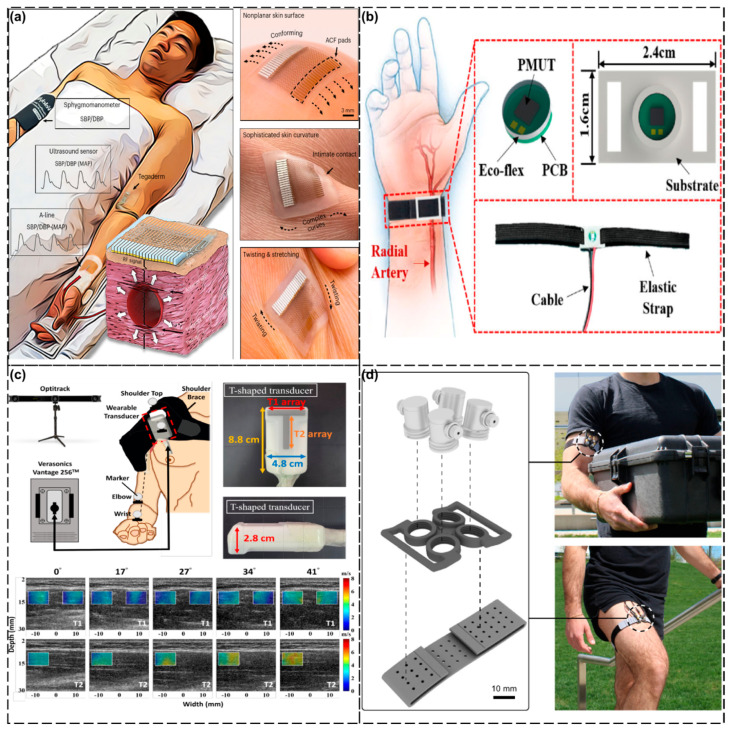
Wearable ultrasound detection in the limbs. (**a**) Schematic of the testing setup and the working principle of the device. Reproduced with permission: Copyright 2024, Springer Nature [128]. (**b**) Schematic of the wearable device with close-up details of the device construction. Reproduced with permission: Copyright 2023, IEEE [129]. (**c**) Wearable transducer attached on the shoulder with shoulder brace. Reproduced with permission: Copyright 2024, IEEE [130]. (**d**) The A-mode ultrasound system. Reproduced with permission: Copyright 2024, Springer Nature [131].

We have presented many examples of blood pressure measurement method above, but the most commonly used blood pressure measurement method in daily life is the traditional cuff blood pressure device [141]. Although this device is widely used in clinical applications, its intermittent measurement mode has significant limitations, and frequent inflation and deflation not only interferes with the patient’s daily activities, but it also affects the quality of sleep. To address this clinical pain point, Zhou’s research team [128] proposed a breakthrough solution: an ultra-thin flexible ultrasound transducer. The device adopts a structural design with an overall thickness of only about 800 microns, which can perfectly fit the curved surface of the skin of various parts of the human body and realize non-invasive and continuous blood pressure monitoring. Experimental data from multiple trials has demonstrated that this sensor has excellent biocompatibility and mechanical durability. It can still maintain functional integrity after sterilization treatment, fully meeting the clinical needs of long-term wearable monitoring. All the studies were approved by the Ethics Review Committee of the University of California, San Diego. All the participants signed an informed consent form, ensuring the ethics of the study. This device requires more improvements. For instance, the performance of the sensor may be severely affected by arrhythmias, leading to deviations in the measurement results. Physiological differences among different populations (such as vascular stiffness) may affect the measurement results. In some cases, such as patients in an intensive care unit, more frequent recalibration may be required (Figure 6a). Further, Zhou’s team [129] reported a more advanced blood pressure monitoring system. This system employs a 23 × 26 array aluminum nitride (AlN) Piezoelectric Micromachined Ultrasonic Transducer (PMUT) to achieve continuous and real-time monitoring of blood pressure waveforms. Validation experiments were conducted in in vitro arterial models and human radial arteries. The results showed that the measurement error was within 5 mmHg, demonstrating excellent accuracy, stability, and repeatability. It is particularly noteworthy that the system could maintain measurement stability under dynamic conditions, such as when the hand is moving, while ensuring wearing comfort. However, there is a very crucial issue with this device that has not been resolved. Blood pressure measurements are affected by the position of the radial artery relative to the heart. For instance, the measurement value decreases when the arm is raised, while it increases when the arm is lower than the heart. This indicates that in practical applications, the influence of body position on the measurement results needs to be considered (Figure 6b).

Muscles make up a large portion of our limbs and are an integral target for monitoring physiological signals. For example, shoulder muscle injuries are very important in current medical research and clinical practice because they not only affect the quality of the daily life of the patient, but can also lead to long-term dysfunction [142]. Current treatments include conservative treatments (e.g., anti-inflammatory drugs and physical therapy) and surgical treatments (e.g., arthroscopic surgery), but the effectiveness of these methods depends on an accurate assessment of the extent of muscle and tendon damage. For this reason, Chen et al. [130] investigated and developed a wearable dual-directional shear wave elastography (DDSWE) device that is capable of measuring both longitudinal and transverse shear wave velocities (SWVs) during shoulder muscle movement, thus providing dynamic information on muscle mechanical properties. It was tested on research subjects who were healthy volunteers without a specific history of diseases. A total of five male volunteers were recruited for the experiment, with an average age of 23.2 years and an average BMI of 22.4 kg/m^2^. All the participants completed the same movement under the same conditions, that is, performing shoulder abduction movements within the range of 0° to 60°. During this movement, the average lateral and longitudinal SWVs increased from 2.24 m/s to 3.35 m/s and from 2.95 m/s to 5.95 m/s, respectively, which were in line with the expectations and proved the effectiveness of the equipment. The size of this device may limit its application in certain shoulder areas, especially for those with irregular shoulder shapes. This device may become an important tool for the diagnosis and rehabilitation assessment of shoulder diseases, providing clinicians with more accurate mechanical characteristic data (Figure 6c).

Mechanical loads refer to the forces or torques applied to biological tissues (e.g., muscles, bones, tendons, etc.), which can be internally generated (e.g., forces generated during muscle contraction) or externally applied (e.g., weight of an object or external resistance) [143]. Quantifying joint torque (a form of mechanical loading) during dynamic activities can provide new insights into muscle function and exercise biomechanics, with potential applications in injury prevention and rehabilitation. Jin et al. [131] developed an A-mode ultrasound wearable monitoring tool, whereby A-mode ultrasound can efficiently track changes in muscle thickness and be used to estimate joint torque during different dynamic activities. The author adopted a multi-stage experimental design, including laboratory tests and real-world application tests, to verify the method of estimating joint torque based on A-mode ultrasound. This design ensures the validity and reliability of the experimental results by gradually increasing the complexity and number of variables. First, isokinetic contraction tests were conducted in a controlled environment to establish a model and verify its accuracy. Secondly, the practicality of the model was further verified through dynamic functional activities such as dumbbell curls and outdoor walking. During the isokinetic contraction process, the torque estimation error of the elbow and knee was less than 7.6%, and the coefficient of determination (R^2^) was greater than 0.92. In the practical application scenarios such as dumbbell curls and outdoor walking, the A-mode ultrasonic system accurately estimated the torque changes at the elbow and knee, verifying its feasibility during unconstrained dynamic activities. Currently, the research uses joint torque rather than muscle force as the benchmark, and the direct non-invasive measurement of muscle force remains challenging. At this stage, the focus is mainly on superficial muscles. Further research is needed on deep muscles and in complex joints. It is recommended to combine feature extraction methods (such as MBTA) with advanced machine learning algorithms to improve the accuracy of torque estimation. At the same time, applying multi-sensor fusion technology to the same muscle or different muscles should be considered to enhance the robustness and application scope of the system (Figure 6d).

In summary, in the field of limb monitoring, new wearable ultrasound technology has achieved significant breakthroughs and has advantages over traditional devices. For blood pressure monitoring, the ultra-thin flexible ultrasonic sensor (800 microns thick) and the aluminum nitride piezoelectric array system (23 × 26 PMUT) have addressed the pain point of intermittent measurements in cuff-type devices, achieving continuous dynamic monitoring with an accuracy of ±5 mmHg that is free from motion interference. In terms of muscle assessment, the bidirectional shear wave elastography (DDSWE) device has broken through the limitation of traditional elastography where it cannot assess the mechanical characteristics of muscles in motion in real time by dynamically measuring the shear wave velocity. The A-mode ultrasound system can provide dynamic estimation of joint torque with an error of less than 7.6%, overcoming the drawback of traditional biomechanical detection, which can only be used in laboratory environments.

## 5. Conclusions

This paper provides a systematic overview of the latest research on wearable ultrasound detection devices, focusing on summarizing their principles, structural designs, and some typical applications. In the field of medical diagnosis, flexible wearable ultrasound detection devices show comparable performances to those of commercial ultrasound probes, especially on irregular skin surfaces such as the chest and neck, and can provide more comprehensive continuous monitoring and imaging information, whether at rest or during movement. These devices can effectively capture physiological signals, expanding the scope of ultrasound detection applications.

Although wearable ultrasound detection devices show good potential for development, they still face a series of challenges in clinical applications. The first is the optimization of the coupling material. The current coupling material is prone to drying and separation from the edge of the transducer during use, which affects the transmission efficiency of the ultrasound signal. Therefore, coupling materials with excellent long-term moisturizing properties and strong adhesion need to be developed in the future. Such materials should not only be stable in humid environments, but also have good biocompatibility to adapt to the skin of different patients. Secondly, the piezoelectric materials need to be improved. The existing piezoelectric materials are still deficient in terms of frequency response and acoustic impedance matching. As the size of the vibrating element shrinks, the performance of conventional materials may not be able to meet the increasingly stringent detection requirements. Therefore, future research should focus on reducing acoustic impedance, improving transverse vibration suppression, and developing new piezoelectric materials. These improvements will help to increase the effective transmission of acoustic waves, which in turn will enhance imaging quality and monitoring accuracy. For the wearable ultrasonic testing system, merely designing the structure of the transducer is far from enough. We need an entire system that is capable of processing data and providing energy. The ultrasonic information to be processed is extremely vast, which requires us to not only innovate the structure and materials, but also to put more effort into hardware design and signal processing. We also have to address the problem of integrating multifunctional sensors. Combining ultrasound technology with chemical and biological sensors to realize the simultaneous monitoring of multiple physiological characteristics is the key to comprehensive health monitoring. Future devices should have multifunctional sensing capabilities and be able to collect data without interference. For example, the integration of oxygen saturation, heart rate, and even blood glucose monitoring can dramatically increase the clinical application value of the device. Of course, there is still the problem of heat dissipation that needs to be solved. The piezoelectric element of a flexible transducer is usually wrapped in a flexible material and lacks the air back-symmetric heat dissipation space of a conventional transducer, and prolonged ultrasound testing may lead to overheating of the device, which may affect its durability and performance. Therefore, the development of effective heat dissipation strategies, such as the use of materials with excellent thermal conductivity or the integration of heat dissipation channels, should be the focus of future research.

With the continuous advancement of flexible electronics, materials science, and micro-nano fabrication technology, the future of flexible wearable ultrasound detection devices is promising. These devices can not only be applied to clinical monitoring, but could also play an important role in the fields of sports medicine, elderly care, and family health management. By combining with artificial intelligence and big data technology, future devices are expected to achieve real-time data analysis and intelligent feedback, providing support for personalized medicine. In conclusion, although flexible wearable ultrasound detection devices still face many challenges in terms of technology and application, through continuous research and innovation, these devices have the potential to revolutionize traditional medical monitoring and provide patients with more convenient and efficient health-management solutions.

## Figures and Tables

**Table 1:** Summary table of designs and preparation methods for flexible ultrasonic transducers.

Component	Materials/Techniques	Advantages	Challenges/Limitations	Ref(s).
Piezoelectric Elements	Rigid materials: PZT ceramics	High piezoelectric coefficients (PZT)	Low piezoelectric constants (ZnO/AlN)	[78,79]
	Flexible materials: ZnO/AlN thin films, PVDF copolymers	Flexibility (films/polymers)	PVDF has poor coupling	[80,81,82]
	Hybrid: PZT + serpentine hinges	Hybrid balances performance and flexibility	Array spacing trade-off (crosstalk vs. stretchability)	[83,84,85]
Interconnect Electrodes	Solid metals (Ag paste, Cu)	High conductivity (metals)	Solid metals fracture under strain	[85,86]
	Liquid metals (AgNWs, EGaIn)	Stretchability (liquid metals)	Liquid metals require complex processing	[87,88,89]
	Design: Serpentine “island-bridge” layout	Serpentine structure enhances ductility		[90,91]
Flexible Substrate	PDMS, Ecoflex, PU, hydrogels	Biocompatibility (PDMS)	PDMS has poor adhesion	[92,93,94,95]
	Treatments: UV/plasma activation	Hydrogels offer bioadhesion	Hydrogels dehydrate over time	[94,95]
	Reinforcements: CNTs, graphene	High elasticity (Ecoflex/PU)		[96,97]
Flexible Encapsulation	Hydrogel–elastomer composites	Acoustic impedance matching	Conventional hydrogels harden when dry	[98,99]
	Silicone elastomers	Long-term adhesion (48 h)		[100,101]
		Acoustic impedance matching		

**Table 2:** Summary of wearable ultrasonic monitoring equipment applications.

Anatomical Region	Clinical Focus	Key Technology/Device	Advantages	Ref.
Head and Neck	Cerebral hemodynamics	Low-frequency (2 MHz) ultrasound patch with copper mesh shielding	- Can penetrate cranial windows - SNR improvement of 5 dB - Motion-resistant long-term monitoring	[120]
	Carotid blood pressure (ABP)	Flexible phased-array transducer (39 × 37 × 0.71 mm)	- Non-invasive, continuous monitoring - Matches invasive arterial catheter accuracy	[121]
	Broad tissue monitoring	Braidable polymer ultrasound transducer (PUT) with 93% bandwidth (3.68–10.08 MHz)	- Stable after 10,000 deformations - SNR comparable to commercial probes	[122]
Thoracic Cavity	Cardiac function	Wearable cardiac ultrasound array + liquid metal electrodes + deep learning algorithms	- Real-time multi-angle LV imaging - Automated metrics (e.g., ejection fraction)	[75]
	Exercise hemodynamics	Fully integrated system (USoP) with M-mode imaging	- Capable of 12-h monitoring during motion - Tracks carotid pulsations during head rotation	[123]
	Breast cancer screening	cUSBr-Patch with Yb/Bi-doped PIN-PMN-PT single-crystal phased array	- Wider field of view (30 mm depth) - Operator-independent imaging	[124]
Abdominal Cavity	Acute Liver Failure (ALF)	Bioadhesive Ultrasound Shear Elastography (BAUS-E)	- Real-time liver stiffness tracking - Non-invasive disease progression monitoring	[125]
	Bladder volume (LUTD)	UBVM system with multipoint reflectance + spherical fitting algorithms	- Average error of 11.17% - Wireless continuous monitoring	[126]
		cUSB-Patch (5-array design)	- Multi-angle imaging without probe movement - Error: 3.2–10.8%	[127]
Limbs	Continuous blood pressure	Ultra-thin flexible transducer (800 μm)	- Curved-surface conformability - Sterilization-resistant, long-term wear	[128]
	Shoulder muscle injuries	AlN PMUT array (23 × 26)	- Accuracy: ±5 mmHg - Dynamic condition stability (e.g., hand movement)	[129]
	Joint torque quantification	Dual-directional shear wave elastography (DDSWE)	- Measures longitudinal/transverse SWVs during motion	[130]
		Wearable A-mode ultrasound system	- Torque error of <7.6% during dynamic activities (R^2^ > 0.92)	[131]

## Data Availability

No new data were created or analyzed in this study.
